# Omega-3 Index as a Sport Biomarker: Implications for Cardiovascular Health, Injury Prevention, and Athletic Performance

**DOI:** 10.3390/jfmk9020091

**Published:** 2024-05-22

**Authors:** Alessandro Medoro, Andrea Buonsenso, Marco Centorbi, Giuseppe Calcagno, Giovanni Scapagnini, Giovanni Fiorilli, Sergio Davinelli

**Affiliations:** Department of Medicine and Health Sciences “V. Tiberio”, University of Molise, 86100 Campobasso, Italy; alessandro.medoro@unimol.it (A.M.); andrea.buonsenso@unimol.it (A.B.); marco.centorbi@hotmail.it (M.C.); giuseppe.calcagno@unimol.it (G.C.); giovanni.scapagnini@unimol.it (G.S.); sergio.davinelli@unimol.it (S.D.)

**Keywords:** omega-3 polyunsaturated fatty acids, EPA, DHA, dietary supplementation, sports, exercise, elite and non-elite athletes, performance, cardiovascular health, injury prevention

## Abstract

The composition of polyunsaturated fatty acids (PUFA) in the cell membrane plays a crucial role in cell signaling and function. Physical activity can induce shifts in PUFA metabolism, potentially altering their membrane composition. Given the multifaceted regulatory and structural roles of PUFA, training-related fluctuations in PUFA concentrations may impact health and athletic performance in both elite and non-elite athletes, highlighting the critical role of these fatty acids’ nutritional intake. The ω-3 index (O3I), a biomarker reflecting the proportion of eicosapentaenoic acid (EPA) and docosahexaenoic acid (DHA) in red blood cell membranes, is considered a marker of cardiovascular risk, gaining increasing interest in sports medicine. Dietary interventions aimed at maintaining an optimal O3I may offer several benefits for elite and non-elite athletes, including cardiovascular health performance optimization, recovery, and injury prevention. Here, we discuss emerging evidence on the application of O3I in sports and physical exercise, highlighting its promising role as a biomarker in a wide range of sports practices.

## 1. Introduction

Fatty acids (FA) are essential components of cells, crucial for creating an optimal environment for membrane protein function. The length and degree of membrane unsaturated FA in cell membranes are the main determinants of membrane fluidity, cell signaling, and overall cellular function [1]. The composition of FA in cell membranes not only reflects dietary fat consumption but is also influenced by FA metabolism and many other factors [2,3]. Physical activity and sports can lead to metabolic changes in the utilization of FA as energy sources, the mobilization of FA reserves in adipose tissue, and the transport of lipids between organs and tissues [4]. Long-term and intense physical training can lead to changes in FA membrane composition, particularly in long-chain omega-3 (ω-3) and omega-6 (ω-6) polyunsaturated FA (PUFA), influencing cellular processes and physiological functions [5,6].

**Figure 1:**
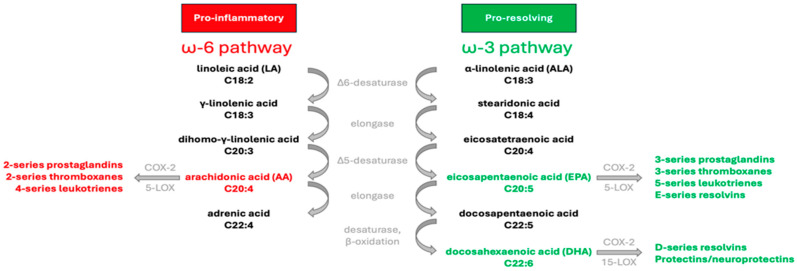
Biosynthesis and metabolism of principal ω-3 and ω-6 PUFA. Linoleic acid (18:2; LA) and α-linolenic acid (18:3; ALA) are essential PUFA acquired from dietary sources. Through a series of desaturation and elongation reactions, mammals convert LA and ALA into long-chain PUFA. Important intermediates involved in synthesizing eicosapentaenoic acid (20:5; EPA), docosahexaenoic acid (22:6; DHA), and arachidonic acid (20:4; AA) include stearidonic acid (18:4), eicosatetraenoic acid (20:4), γ-linolenic acid (18:3), and dihomo-γ-linolenic acid (20:3). AA and EPA serve as substrates for generating various eicosanoids, including prostaglandins, thromboxanes, and leukotrienes, which play pivotal roles in regulating inflammatory processes. DHA undergoes metabolism to form resolvins and protectins, which actively contribute to resolving inflammation.

Although they share the same enzymes, there are two distinct pathways for the synthesis of the long-chain ω-3 and ω-6 PUFA, as illustrated in Figure 1. Humans are unable to produce the precursors of these FA families, linoleic acid (18:2ω-6; LA) and α-linolenic acid (18:3ω-3; ALA). The consumption of these essential FA through diet facilitates the synthesis of arachidonic acid (20:4 ω-6; AA), eicosapentaenoic acid (20:5ω-3; EPA), and docosahexaenoic acid (22:6ω-3; DHA) involving elongation and desaturation reactions. These compounds play crucial roles in regulating various homeostatic processes by influencing the production of bioactive signaling lipids known as eicosanoids. However, ω-3 and ω-6 PUFA exert contrasting effects on metabolic functions. Cyclooxygenases-2 (COX-2) synthesizes prostaglandins (PGs) and thromboxane (TXA) of the two-series (A2, E2, I2, and TXA2) from AA, while lipoxygenases (5-LOX) act on AA to produce leukotrienes (LTs) of the four-series (B4, C4, and E4). These eicosanoids derived from AA play various roles in physiological processes, such as promoting inflammation, platelet aggregation, vasoconstriction, and immune responses [7]. Through a similar series of reactions metabolized by the same enzymes, ALA transforms into EPA, from which COX-2 and 5-LOX can metabolize to produce PG and TXA of the three-series (B3, D3, E3, I3, and TXA3), and LT of the five-series (B5, C5, and D6), respectively. The biosynthesis of DHA involves further reactions, including elongation, desaturation, and β-oxidation. DHA can then be metabolized into autacoids like D-series resolvins (RVD1 to RVD6) and protectins (neuroprotectin D1). Mediators derived from EPA and DHA exhibit potent anti-inflammatory properties and act as specialized agents crucial for resolving inflammation [8].

**Figure 2:**
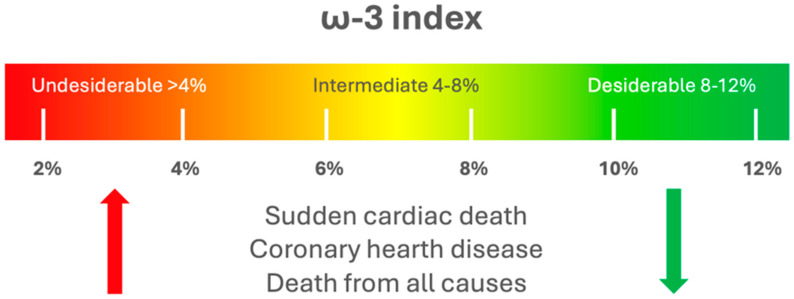
O3I risk thresholds. A protective target level for O3I was identified at approximately 8%, while levels below 4% were associated with an elevated risk of cardiovascular diseases.

Different methodologies are available for quantifying PUFA levels and their mediators. Common methods include enzyme-linked immunosorbent assays (ELISA) and radioimmunoassays. However, these techniques are limited as they can only measure one metabolite at a time, lack complete selectivity, are prone to cross-reactivity, and apply only to specific lipid types. The latest progress in mass spectrometry (MS) has facilitated the advancement of lipidomics, enabling the concurrent qualitative and quantitative evaluation of a wide array of lipid species, including PUFA. Liquid chromatography (LC)-MS/MS is particularly powerful for PUFA analysis, offering high sensitivity and specificity in the evaluation of a wide array of lipid species [9]. Due to their stability, PUFA indices are often measured in the red blood cell (RBC) membrane, representing the preferred sample type. It exhibits lower variability compared to measurements in plasma, primarily due to the limited exchange between plasma and cells and the incorporation of dietary EPA and DHA in a dose- and time-dependent manner [10,11,12]. However, this method is invasive and costly, requiring complex sample handling. Dried blood spot (DBS) sampling, usually obtained from the fingertip, offers a simpler alternative, allowing for minimally invasive sample collection and easy acceptance by patients. DBS has been validated for PUFA analysis in various studies, demonstrating good correlation and low variability compared to traditional RBC measurements. Despite some limitations, DBS holds promise for large-scale studies and may be suitable for estimating PUFA indices in clinical settings [9,13,14].The concentration of PUFA in blood lipids is commonly used to measure dietary intake and estimate their levels in cell membranes [15]. PUFA levels in whole blood (WB) and RBC mirror the PUFA composition in the cellular membranes of the major organs and tissues [16,17]. The ω-3 index (O3I) is defined by the amount of EPA + DHA in RBC membranes expressed as a percentage of the total RBC membrane FA. O3I was initially proposed as a marker to study the risk of death from coronary heart disease (CHD) [18]. The recommended protective level for O3I is approximately 8%, while levels below 4% are linked to a higher risk of disease (Figure 2). Nevertheless, these thresholds have not yet been confirmed in extensive human studies [9].

Indeed, nowadays, O3I is considered a “low-noise” parameter suitable for use in epidemiological and clinical studies, as it is not affected by acute ω-3 PUFA intake or severe clinical events [12,19,20]. Although specific guidelines on an O3I cut-off point have not been established, the recommended target range for athletes is currently set at 8–11% [21]. Various factors, including training intensity and volume or body weight, can impact O3I percentages. However, the influence of metabolic status and training level on the response to supplementation requires further investigation.

Due to their diverse regulatory and structural functions, training-related changes in PUFA concentrations may impact global health and athletic performance in both elite and non-elite athletes, highlighting the critical importance of the adequate intake of PUFA and suggesting their supplementation as a strategy to maintain optimal levels [22,23,24]. Dietary supplementation with ω-3 long-chain EPA and DHA has been shown to reduce the production of inflammatory compounds in athletes participating in high-intensity and long-duration exercise, such as marathon or triathlon competitions [6,25]. The anti-inflammatory properties of ω-3 long-chain PUFA are thought to be due to EPA’s inhibition of eicosanoid synthesis from ω-6 long-chain PUFA arachidonic acid (AA; 20:4ω-6) [26].

Moreover, several studies have assessed the efficacy of ω-3 long-chain PUFA supplementation on oxidative stress, muscle damage, and inflammation during exercise [27]. However, the evidence suggests that benefits are not limited to reducing inflammation and promoting faster recovery but also contribute to overall cardiovascular health. Given the demanding nature of athletic training and competition, optimizing these physiological aspects becomes paramount for athletes aiming to perform at their peak and maintain long-term well-being. Integrating EPA and DHA supplementation into their nutritional strategy could provide a valuable edge in enhancing athletic performance and supporting overall health. Therefore, in this review, we describe the most recent evidence of the role of O3I in elite and non-elite athletes, discussing its potential as a sports biomarker in cardiovascular health, injury prevention, and athletic performance. We then discuss optimal O3I levels, dosages, and timing strategies for ω-3 PUFA supplementation.

## 2. ω-3 Index and Cardiovascular Health in Sports

Cardiovascular health plays a vital role in optimizing sports performance. Improvements in resting heart rate and muscle blood flow can enhance performance and aid in quicker recovery after exercise by facilitating the exchange of waste products and nutrients [28]. The relationship between circulating levels of ω-3 PUFA and cardiovascular health, as well as the risk of cardiovascular diseases (CVD), is a topic of significant discussion. ω-3 PUFA can decrease the risk of CVD by improving membrane phospholipids with EPA and DHA and promoting anti-atherosclerotic, anti-inflammatory, and plaque-stabilizing effects. Therefore, numerous studies have suggested O3I as a valuable clinical biomarker for assessing cardiovascular risk, even in athletes, due to its strong correlation to EPA and DHA levels in cardiac tissue (Table 1) [29,30].

In particular, a cut-off of <4% was proposed to identify individuals at high risk of CVD and >8% to identify individuals at low risk [31]. However, studies examining the dietary habits of athletes have shown that most do not meet the recommended intake levels for essential nutrients, including EPA and DHA. A study of 404 Division I football players found that none had an O3I > 8%. Low O3I has also been identified in almost all 106 German elite winter endurance athletes studied. This deficiency ω-3 PUFA is reflected in an increased risk of mortality and cardiovascular events [32,33,34].

In a recent meta-analysis of interventional trials, supplementation with EPA and DHA was described as an effective lifestyle strategy for CVD prevention, and the protective effect is probably increased with dosage [35]. Even a 1% increase in O3I can lead to a 58% reduction in the risk of ventricular fibrillation in patients with cardiac arrest [36]. Consuming fish meals once a week may reduce the risk of CHD by 15%, with a dose–response relationship showing a 40% risk reduction with higher intake (5 times a week) [37]. Similar results were seen for stroke [38]. An intake of 850 mg of EPA + DHA for 3.5 years decreased the risk of sudden death by 45% in post-myocardial infarction individuals [39]. A recent review suggests that non-elite athletes may benefit more from ω-3 PUFA supplementation and may require shorter durations to experience the benefits compared to elite athletes [40]. Drobnic et al. evaluated the effects of two different dosages of ω-3 PUFA (760 mg/day or 1140 mg/day) in 24 athletes from a summer sports federation over a 4-month intervention period. Findings revealed a dose-dependent increase in the O3I, with athletes starting at lower initial levels showing a more pronounced response to supplementation [41]. Moreover, Flock et al. reported that individuals with lower body weight respond more favorably to ω-3 PUFA intake and reach the target O3I more quickly. The authors proposed specific daily ω-3 PUFA intake levels based on body weight to achieve an O3I within the optimal range [42]. These findings contrast with the results of a study in an elite athlete population, where variations in O3I were not observed based on athletes’ body weight, likely due to alignment with the demands of their sport [41].

Football players and runners are the two categories principally studied regarding O3I levels and their correlation with cardiovascular risk. Indeed, a significant percentage of football athletes may be at high risk of CVD, with supplementation of ω-3 PUFA leading to improvements in O3I levels and reduced risk of CVD. In detail, 34% of them were at high risk of CVD (O3I < 4%), 66% were at moderate risk (O3I between 4% and 8%), and no athlete was at low risk (O3I > 8%) [34]. Similarly, DHA-rich algal oil supplementation for 5 weeks improved both the low baseline O3I and high AA/EPA ratio among Division I American college football players with body mass-specific dose effects [43]. Findings from a study of 27-week supplementation with a placebo or 2, 4, or 6 g/day of DHA to 69 American-style football players demonstrated a dose–response incorporation of DHA into RBC membranes up to 6 g/day. Furthermore, this supplementation can be used to rapidly achieve the desired O3I (>8%) in athletes in only 8 weeks, reducing the risk of CVD [44]. Overall, 5-week supplementation with 6 g/day of DHA has been shown to improve cardiovascular function and reduce the risk of CVD in Australian rules for football players [45].

Recreational marathon runners supplemented with ω-3 PUFA for 3 weeks have been shown to experience positive changes in the RBC lipid composition, serum adipocytokines, and post-exercise proinflammatory cytokine levels, further supporting the cardio-protective benefits of increased O3I levels and the reduced AA/EPA ratio [46]. Similarly, distance runners supplemented for three weeks with ω-3 PUFA showed increased ω-3 PUFA content in the RBC membrane and lower blood concentrations of cardiac damage markers and inflammation mediators, suggesting an improvement in cardiovascular function [47]. Additionally, 3 weeks of supplementation with ω-3 PUFA in recreational runners have also shown promising results in significantly improving O3I (<8%) and reducing the AA/EPA ratio and, consequently, cardiovascular risk. However, longer trial durations may be necessary to achieve optimal O3I levels [46].

**Table 1:** Interventional and observational studies in which the levels of O3I in elite or non-elite athletes were assessed.

First Author, Year	Population	Study Design	Intervention with Dosage	Key Findings
Anzalone et al., 2019 [34]	404 NCAA Division I football players	Retrospective, cross-sectional study	N.A.	- Low O3I in 34% of athletes - >8% O3I in no athletes
Davinelli et al., 2019 [48]	257 non-elite runners	Retrospective, observational study	N.A.	- Inverse correlation between O3I and AA/EPA ratio - Gradual decrease in the O3I and increase in AA/EPA ratio with higher weekly running distance
Davinelli et al., 2023 [49]	275 non-elite runners	Retrospective, observational study	N.A.	- Association of high values of O3I with the lowest number of running-related injuries
Drobnic et al., 2017 [41]	24 summer sports athletes	Randomized, parallel-group study	Supplementation with 760 mg/day or 1140 mg/day ω-3 PUFA for 4 months	- Dose-dependent increase in the content of EPA and DHA in the red blood cells at 4 months - Greater increment in O3I in athletes with lower basal levels
Heileson et al., 2021 [50]	66 NCAA American football athletes	Multi-site, non-randomized, parallel-group study	Supplementation with 2000 mg DHA, 560 mg EPA, or 320 mg DPA 4 times per week for a total of 89 days	- Increase in O3I and reduced elevation in serum NF-L levels after supplementation
Hingley et al., 2017 [51]	26 trained male subjects	Double-blind, placebo-controlled study	Supplementation with 560 mg DHA and 140 mg EPA/day for 8 weeks	- Increase in O3I and reduced relative oxygen consumption during the cycling time trial after supplementation
Jaworska et al., 2023 [47]	24 male long-distance runners	Randomized, placebo-controlled study	Supplementation with 3 g of ω-3 PUFA for 3 weeks	- Improvement in blood lipid profiles and O3I - Reduction in inflammation mediators and cardiac damage markers after the eccentric exercise tests
Larkin et al., 2024 [43]	47 American college football players	Longitudinal and cross-sectional study	Supplementation with algae oil each weekend for 5 weeks (equivalent to 750 mg of DHA and 375 mg EPA per day)	- Improvement in both low baseline O3I and high AA/EPA ratio with body mass-specific dose effects
Lembke et al., 2014 [52]	69 male and female college students	Randomized, placebo-controlled study	Supplementation with 2.7 g ω-3 PUFA/day for 30 days	- Less pain related to DOMS following heavy exercise at 72 and 96 h in subjects with a higher O3I reported - Lower serum levels of blood lactate in subjects with a high O3I - Reduction in CRP at 24 h in high O3I subjects
Lust et al., 2023 [44]	69 American football players	Randomized, double-blind, placebo-controlled, parallel-group study	Supplementation with 2, 4, or 6 g/day of DHA for 27 weeks	- Dose–response incorporation of DHA into RBC membranes up to 6 g/day - Achievement of >8% O3I in athletes in 8 weeks with 6 g/day of DHA supplementation
MacArtney et al., 2014 [53]	39 physically fit and healthy males	Double-blind, parallel-group study	Supplementation with 140 mg of EPA and 560 mg of DHA/day for 8 weeks	- Increase in O3I, reduction in mean heart rate during exercise, and improved heart rate recovery after supplementation
Tomczyk et al., 2023 [54]	26 amateur male long-distance runners	Randomized, parallel-group study	Supplementation with 2234 mg of EPA and 916 mg DHA/day for 12 weeks	- Increase in O3I and indicators of running performance, including running economy and peak oxygen uptake
Zebrowska et al., 2021 [46]	24 recreational marathon runners	Randomized, blind, placebo-controlled study	Supplementation with 852 mg EPA, 1602 mg DHA, and 12 mg and 30 µg of vitamin E and D/day for 3 weeks	- Increase in O3I and decrease in AA/EPA ratio after supplementation - Positive changes in lipid composition of erythrocytes, serum adipocytokines, and post-exercise proinflammatory cytokine levels

AA, arachidonic acid; CRP, c-reactive protein; DHA, docosahexaenoic acid; DOMS, delayed onset muscle soreness; DPA, docosapentaenoic acid; EPA, eicosapentaenoic acid; N.A., not applicable; NCAA, National Collegiate Athletic Association; O3I, ω-3 index; RBC, red blood cells.

The O3I emerges as a promising biomarker for evaluating the susceptibility of athletes to cardiac events, encompassing ventricular and atrial fibrillations, as well as sudden cardiac arrest in CHD, facilitating the stratification of individuals at risk. Moreover, it provides valuable insights into baseline ω-3 PUFA levels, thereby enabling targeted interventions, such as recommending an increase in ω-3 PUFA intake. This underscores its potential significance in clinical practice for optimizing cardiovascular health in athletes.

## 3. ω-3 Index and Sports Injury Prevention

Each year, an estimated 3 to 5 million sports injuries occur, likely due to the demanding competitive schedule and intense training regimens associated with sports [55,56,57]. The majority of these injuries affect the musculoskeletal system, accounting for approximately 40%, with a focus on hamstring muscles, ligaments, and joints. Nutritional strategies may help prevent some of these injuries by protecting muscle tissue, regulating the immune system, and improving inflammation processes [58,59,60]. Indeed, an imbalance in inflammation levels (high acute inflammation status or its excessive reduction) can hinder muscle recovery and lead to pain and temporary loss of function [61].

Optimal ω-3 PUFA levels, principally EPA and DHA, have been shown to effectively modulate the inflammatory process by reducing the levels of classic inflammatory and injury markers, including prostaglandin E2, interleukin-6 (IL-6), tumor necrosis factor α (TNF-α), lactate dehydrogenase (LDH), and creatine kinase (CK), pain, and oxidative stress responses to physical exercise. While some studies suggest that inflammation plays a role in triggering physiological processes and inhibiting it may hinder muscle tissue regeneration, the overall benefits of ω-3 PUFA in managing excessive inflammation are evident. Supplementation with 1.8 g/day of ω-3 PUFA led to a decrease in inflammatory markers associated with muscle damage 24 and 48 h following eccentric training in untrained men [62,63]. This suggests that ω-3 PUFA may negatively influence not only the biosynthesis of ω-6-derived pro-inflammatory mediators but also the signaling pathways that regulate gene expression in inflammatory cells, reducing cellular damage induced by physical exercise. A plausible explanation for these effects is their impact on the nuclear factor kappa B (NF-κB) system. NF-κB serves as a key transcription factor that is responsible for up-regulating genes encoding proteins involved in inflammation, including various cytokines, adhesion molecules, and COX-2 [8]. Consistent with this, EPA or fish oil has been shown to decrease lipopolysaccharide (LPS)-induced activation of NF-κB in human monocytes. Similarly, DHA has been found to reduce NF-κB activation in response to LPS in cultured macrophages and dendritic cells [64,65,66,67,68,69].

Multiple studies have demonstrated that positive changes in O3I may mitigate traumatic brain injury (Table 1). This was shown by analyzing serum neurofilament light (NF-L) levels as a surrogate marker for head trauma in American football players, a sport with the highest incidence of traumatic brain injury [67,68,69]. A reduction in DHA levels in the brain occurs as a result of traumatic brain injury, leading to slower recovery of motor function, increased anxiety behaviors, and cognitive deficits [70,71,72,73]. Indeed, DHA has a crucial role in the central nervous system where it is highly concentrated, being 100 times more abundant than EPA [74]. Oliver et al. conducted a study on 81 American football athletes during a competitive season, evaluating the effects of different daily doses of DHA (2, 4, and 6 g). The results showed that DHA supplementation, regardless of the dose, helped reduce traumatic brain injury damage, with 2 g/day being sufficient to reduce serum NF-L levels [75]. Similarly, Heileson et al. analyzed the impact of 2 g/day of ω-PUFA on 31 American football players throughout a regular season compared to a control group that did not receive supplementation. The supplementation group showed an increase in O3I and a reduced elevation in serum NF-L levels. In contrast, the control group experienced a significant increase in serum NF-L compared to the baseline [50]. Considering the elevated serum NF-L levels found in other contact sports, ω-3 PUFA supplementation may be considered for all athletes participating in sports with a high risk of head trauma [76].

Hudek et al. evaluated the O3I in 29 patients with full-thickness rotator cuff tears. It was observed that patients with rotator cuff tears had a lower O3I compared to those without rotator cuff tendinopathy (5.01% vs. 6.01%). The authors suggested that a lower O3I may indicate increased inflammatory activity in the subacromial space, leading to tendon degeneration [77]. The findings of the inflammatory nature of the subacromial bursa in patients with rotator cuff disease supported this theory [78]. Indeed, in elite and non-elite athletes’ injuries, oxidative stress and inflammation may play a pivotal role in both initial tissue damage and the subsequent repair process. Oxidative stress, characterized by an imbalance between reactive oxygen species (ROS) and nitrogen species (RNS) with antioxidant defenses, can directly damage cellular components and exacerbate tissue injury. Meanwhile, inflammation acts as a double-edged sword, initiating the healing cascade but also contributing to secondary tissue damage if left unchecked [61,79]. Supplementation with approximately 4 g/day of ω-PUFA, which is abundant in EPA and DHA, over 8 weeks demonstrated efficacy in mitigating several parameters associated with oxidative stress and inflammation resulting from acute intense physical exertion [80]. Moreover, higher O3I values were linked to a decreased risk of running-related injuries in recreational runners. Participants who were injured reported an O3I > 4%, indicating O3I as a potential biomarker for assessing running-related injuries [49]. Similarly, Gerlach et al. found a connection between a low daily fat intake and an elevated risk of injuries in competitive runners [81].

## 4. ω-3 Index and Sport Performance

Recent research has suggested that supplementation with ω-3 PUFA may benefit athletes’ performance by reducing inflammation, principally through the inhibition of the COX-2 pathway on ω-6 PUFA, altering cell membrane fluidity, and modifying protein activity and cellular function. Several studies have shown that ω-3 PUFA supplementation and the consequent O3I increase can enhance athletes’ adaptation to training, improving sports performance and supporting post-exercise recovery (Table 1) [82,83,84]. Conversely, various factors, including training volume and intensity, may reduce the O3I levels impacting sports performance. A correlation was demonstrated between distance running training, weekly volume, and a decrease in the O3I of 257 non-elite runners, suggesting that regular running training may negatively contribute to changes in O3I [48].

### 4.1. ω-3 Index and Strength and Power

The role of ω-3 PUFA supplementation in sensitizing muscles to anabolic stimuli is an area of ongoing research, with specific mechanisms still needing to be clarified. Previous findings have suggested that EPA may be the primary anabolic component of ω-3 PUFA compared to DHA. Indeed, in vitro studies have shown that EPA intake can increase muscle protein synthesis and decrease muscle protein breakdown, while DHA intake did not have the same effects [82]. Recent research indicates that ω-3 PUFA may enhance the activities of intracellular signaling molecules and satellite cells involved in maintaining muscle mass [85,86]. Other studies have also suggested that ω-3 PUFA supplementation may increase the protein levels of the mechanistic target of rapamycin (mTOR) and focal adhesion kinase (FAK), which are crucial in regulating muscle protein synthesis and enhancing the muscular response to anabolic stimulation. However, the lack of a control group in this study prevents making conclusive statements about these findings [87].

Smith et al. evaluated the effect of 8 weeks of ω-3 PUFA supplementation (4 g/day). They observed an enhancement of muscle protein synthesis in both young and older adult populations in response to an amino acid intake [88]. Conversely, McGlory et al. did not find improvements in muscle protein synthesis after 8 weeks of 5 g/day of ω-3 PUFA supplementation in response to 30 g of protein ingestion in resistance-trained men. This could be because an optimal protein dose is already sufficient to maximize protein synthesis [85]. The same result was found in a recent meta-analysis of randomized controlled trials, where they did not report a significant improvement in isometric strength following PUFA supplementation. However, all the participants of the included studies in the systematic review were young adults with no adherence to resistance training in the previous 6 months [89]. Furthermore, 4 weeks of supplementation with 0.1 g/kg/day of ω-3 PUFA did not improve strength, power, or speed assessments in competitive soccer players [90].

The paucity of correlation trials in the athlete population between O3I, with or without ω-3 PUFA supplementation, and strength/power represents a notable gap in current research. Further studies are needed to confirm the effectiveness of ω-3 PUFA on the protein synthesis mechanism and to evaluate whether their supplementation is necessary when daily protein intake is adequate. In addition, guidelines regarding the optimal dosage and duration to stimulate these processes need to be clarified. It appears that at least 2 weeks are needed to change the ω-3 PUFA composition of skeletal muscle and enhance anabolic signaling [87]. Moreover, understanding the potential impact of EPA and DHA levels on muscular performance is crucial for optimizing athletic training and recovery strategies. Future studies, including correlation trials and supplementation interventions, are warranted to elucidate the relationship between ω-3 PUFA and strength/power outcomes in athletes. Addressing this gap could provide valuable insights into the role of ω-3 PUFA in enhancing physical performance and optimizing athlete health and well-being.

### 4.2. ω-3 Index and Endurance Performance

Several studies collectively suggest that ω-3 PUFA supplementation and the consequent O3I improvement may have beneficial effects on exercise performance and cardiovascular parameters in different types of athletes. ω-3 PUFA supplementation can lead to a shift towards increased carbohydrate utilization during endurance exercise, resulting in reduced oxygen consumption and perceived exertion. Although EPA is believed to be the primary anabolic component of ω-3 PUFA, DHA may enhance lipid oxidation and insulin sensitivity in muscle cells, thereby improving nutrient permeability. Recent findings suggest that ω-3 PUFA supplementation may increase the expression of the glucose transporter type-4 (GLUT-4) protein, leading to improved insulin sensitivity and potentially enhancing endurance performance.

This shift can improve exercise efficiency by reducing the amount of oxygen needed to resynthesize adenosine triphosphate (ATP) [91,92,93]. Studies have shown that 8 weeks of ω-3 PUFA supplementation can increase EPA and DHA levels in RBC and decrease oxygen consumption and perceived exertion in recreational team sport players during cycling trials [94]. In trained male subjects, supplemented with ω-3 PUFA, increased O3I and reduced oxygen costs during a time cycling trial, although it did not result in improved performance metrics such as test completion time, average power output, or quadricep isometric strength. Further research on athletes is needed to confirm the potential benefits of ω-3 PUFA supplementation on endurance sports performance [51].

In a study conducted on Australian rules on footballer ω-3 PUFA supplementation was given for five weeks, with a daily dosage of 1.56 g of DHA and 0.36 g of EPA. The results showed a significant reduction in heart rate during sub-maximal exercise but no impact on peak heart rate. This suggests that ω-3 PUFA supplementation may decrease heart rate during sub-maximal exercise without affecting maximal exercise performance [45]. Conversely, trained cyclists experienced a significant increase in EPA and DHA in RBC membranes and a decrease in both submaximal and peak heart rates after 8 weeks of supplementation with 3.2 g/day of ω-3 PUFA [95]. Similarly, daily fish oil supplementation led to an increase in O3I, a reduction in mean heart rate during exercise, and improved heart rate recovery in physically fit males without compromising peak heart rate [53].

Moreover, a significant improvement in maximal oxygen uptake (VO_2max_) was observed in endurance-trained athletes after 3 weeks of supplementation with 1.3 g of ω-3 PUFA twice daily. The authors attributed this improvement to an increase in nitric oxide levels, which could potentially impact flow-mediated dilatation and resting artery diameters [96]. Likewise, the effects of 12 weeks of EPA and DHA supplementation (3.1 g/day) in elite long-distance runners improved O3I, and indicators of running performance, including running economy and peak oxygen uptake (VO_2peak_), were determined during a graded exercise test to exhaustion on a treadmill [54]. Lastly, Gravina et al. found that soccer players experienced an improvement in anaerobic endurance running capacity, as measured by the anaerobic endurance capacity (Yo-Yo test) after 4 weeks of supplementation with 0.1 g/kg/day of ω-3 PUFA [90].

### 4.3. ω-3 Index and Exercise-Induced Fatigue

ω-3 PUFA have been shown to aid in the recovery from exercise-induced muscle damage by enhancing the structural integrity of muscle cell membranes and reducing inflammation through the inhibition of the COX-2 pathway and the synthesis of pro-inflammatory molecules [97,98]. Considering that the 96 h following exercise is crucial for optimizing athletic performance [99], various studies have investigated, not without limitations, the impact of ω-3 PUFA supplementation on post-exercise recovery. Jakeman et al. found no significant differences in inflammation markers, such as CK and IL-6, after the acute administration of different dosages of EPA. However, interpreting these findings is challenging as it has been noted that at least 2 weeks of supplementation are required for ω-3 PUFA to be incorporated into muscle tissue [100]. Moreover, the impact of 6 weeks of combined supplementation with 2.8 g/day of ω-3 PUFA s and 30 g of whey protein in competitive soccer players revealed a decrease in plasma CK levels in the 72 h following exercise compared to the group receiving only whey protein supplementation and the control group [83].

The potential effect of ω-3 PUFA and desirable levels of O3I in reducing delayed onset muscle soreness (DOMS) has been a topic of conflicting results in the scientific literature. Indeed, Tartibian et al. administered a 1.8 g ω-3 FA supplement to 27 untrained men, observing a significant decrease in DOMS 48 h post-exercise [101]. Similarly, Jouris et al. found that ingesting 3 g of ω-3 PUFA over 7 days significantly reduced DOMS among 3 male and 8 female participants [102]. Supplementation with 2.7 g of ω-3 PUFA for 30 days resulted in a significant decrease in DOMS at 72 and 96 h post-heavy eccentric exercise in both male and female participants and in healthy young adults afterward. Interestingly, subjects with a higher O3I reported less pain related to DOMS following heavy exercise at 72 and 96 h post-exercise, suggesting that ω-3 PUFA supplementation may improve cell elasticity, thereby reducing muscle damage after physical exercise. This study also reported a decrease in muscle pain and observed lower C-reactive protein (CRP) levels at 24 h and lower blood lactate levels in subjects with higher O3I [52]. More recently, a significant effect of EPA and DHA supplementation was demonstrated in reducing muscle soreness in professional Rugby Union players [103].

Conversely, there have been studies using ω-3 PUFA-rich fish oils supplementation that have not shown any significant differences in DOMS. In total, 4.2 g/day of ω-3 PUFA supplementation 21 days before exercise did not improve exercise-induced muscle damage and delayed-onset muscle soreness (DOMS) with respect to the placebo group after 24 h post-exercise. However, measurements at 48–72–96 h post-exercise, when peak markers may occur, were not evaluated, potentially limiting the exploration of ω-3 PUFA effectiveness in this study [104]. Lenn et al. administered 1.8 g of the ω-3 PUFA supplement over 30 days to 22 subjects but did not observe a significant decrease in DOMS [105]. Similarly, Gray et al. found that supplementation with 3 g of n ω-3 PUFA for 6 weeks did not lead to a significant reduction in DOMS. Notably, they did not identify any discernible differences in DOMS between the experimental and placebo groups [106]. Recently, a systematic review with meta-analysis did not find a clinically significant reduction in post-exercise DOMS following ω-3 PUFA supplementation [89]. Given conflicting data, further randomized controlled studies are needed to clarify the role of ω-3 PUFA supplementation and O3I in reducing DOMS and exercise-induced muscle damage. 

## 5. Conclusions

The O3I holds promise as a biomarker for evaluating the overall health status of athletes. Despite the absence of comprehensive clinical trials, achieving and maintaining O3I levels above 8% is pivotal for reducing cardiovascular risk, mitigating brain and inflammation-induced injuries, and optimizing athletic performance. Given the negative impact of physical activity on EPA and DHA levels, athletes may necessitate higher O3I thresholds compared to the general population. Supplementation with PUFA emerges as a paramount strategy for preserving optimal O3I levels in elite and non-elite athletes. Even short-term supplementation intervals (3–4 weeks) with higher concentrations of PUFA exhibited notable efficacy in increasing O3I levels, thereby conferring benefits on elite and non-elite athletes with very low O3I. However, the reduced number of studies investigating O3I levels in athletes, both with and without supplementation, emphasizes the necessity for additional research aimed at elucidating specific dosages and optimal timing protocols for ω-3 PUFA supplementation for elite and non-elite athletes.

## Data Availability

Not applicable.
